# Patterns and determinants of nutraceutical use and trust mechanisms among adults in Saudi Arabia: a cross-sectional study

**DOI:** 10.3389/fmed.2026.1779602

**Published:** 2026-04-01

**Authors:** Rawan H. Hareeri, Saad M. Wali, Abdulelah A. Alfattani, Ohood K. Almuzaini, Osama K. Alahdal, Nawaf S. Alosaimi, Ashraf A. Alsaedi, Saud A. Hijazi, Malaz J. Gazzaz, Mohammed M. Aldurdunji

**Affiliations:** 1 Department of Pharmacology and Toxicology, Faculty of Pharmacy, King Abdulaziz University, Jeddah, Saudi Arabia; 2 Pharmacology and Toxicology Department, College of Pharmacy, Umm Al-Qura University, Makkah, Saudi Arabia; 3 Pharmaceutical Sciences Department, College of Pharmacy, Umm Al-Qura University, Makkah, Saudi Arabia; 4 Department of Medicine, Ibn Sina National College, Jeddah, Saudi Arabia; 5 Maternity and Children Hospital, Ministry of Health, Makkah, Saudi Arabia; 6 College of Pharmacy, Umm Al-Qura University, Makkah, Saudi Arabia; 7 Pharmaceutical Practices Department, College of Pharmacy, Umm Al-Qura University, Makkah, Saudi Arabia

**Keywords:** consumer trust, dietary supplements, influencer marketing, nutraceuticals, online reviews, pharmacist counseling, Saudi Arabia

## Abstract

**Background:**

Nutraceutical consumption has expanded globally and in the Gulf Cooperation Council (GCC) region, influenced by digital platforms, cultural norms, and preventive health behaviors. In Saudi Arabia, market growth is pronounced, yet little is known about how consumers construct trust and make purchasing decisions in this evolving landscape.

**Aim:**

This study was conducted to evaluate patterns of nutraceutical use, purchasing channels, and trust mechanisms among adults in Saudi Arabia.

**Methods:**

A nationwide cross-sectional online survey was conducted among adults in Saudi Arabia. Of 1,169 responses received, 672 respondents who reported recent nutraceutical use and met the inclusion criteria were included in the analysis. Data collected covered product categories, purchasing frequency, trust determinants, and sociodemographic characteristics. Associations were examined using multivariable regression models.

**Results:**

Vitamins and minerals were most common (554 of 672, 82.4%), followed by probiotics (492 of 672, 73.2%) and botanicals (452 of 672, 67.3%). Purchasing was frequent, with 252 of 672 (37.5%) buying more than once per month. Higher purchasing frequency was associated with very high health consciousness (OR 12.4, 95% CI 6.31–24.8), mid-tier income (OR 2.07, 1.34–3.21), and Northern residence (OR 1.77, 1.11–2.84). Frequent purchasers were more likely to trust online peer reviews (OR 4.34, 2.31–8.22), perceive online and in-store products as equivalent (OR 5.18, 2.80–9.65), and still value pharmacist advice (OR 3.02, 1.65–5.56). Social media was a common discovery route (408 of 672, 60.7%), with 362 of 672 (56.2%) reporting greater trust when influencer content referenced evidence or long-term use. Halal, clinical, or regulatory marks also enhanced confidence (401 of 672, 59.7%). Women reported lower holistic-integration scores, while mid-income groups showed greater responsiveness to influencer cues.

**Conclusion:**

The findings indicate that participants reported relying on both digital sources (online reviews and influencer content) and offline validation (pharmacist advice and quality markers) when making purchasing decisions; initiatives that improve the clarity of product information and professional guidance may support more informed use.

## 1 Introduction

Nutraceuticals and dietary supplements are increasingly integral to preventive health practices worldwide. Global surveys estimate that 40–70% of adults consume these products (1), with motivations often centered on maintaining health, preventing disease, and enhancing wellbeing (2). The COVID-19 pandemic accelerated this trajectory by heightening consumer interest in immune support and self-care strategies (3).

In Saudi Arabia and across the Gulf Cooperation Council (GCC), usage patterns mirror global trends. Approximately 40–47% of adults report supplement use, with vitamins and minerals consistently the leading products (4). Market growth has been rapid, with estimates projecting the GCC dietary supplement sector to reach USD 22 billion by 2025 (5). Rising disposable incomes, a young and health-conscious population, and strong cultural traditions surrounding natural remedies have further propelled this expansion (1).

Trust plays a pivotal role in shaping nutraceutical consumption. Online reviews and influencer marketing increasingly guide product discovery and confidence, yet concerns persist about misinformation and authenticity (6, 7). Parallel to digital influence, cultural and institutional signals such as halal certification and regulatory endorsement remain decisive in consumer acceptance, reflecting both ethical values and expectations of quality assurance (8, 9). Pharmacists contribute additional layers of trust by contextualizing product claims, counseling on safety, and counterbalancing commercial narratives (10). Together, these diverse signals create a hybrid trust architecture that integrates digital, cultural, and professional sources.

Sociodemographic factors further shape engagement. Women generally demonstrate greater use of complementary and alternative medicine, linked to higher health awareness (11), while education and income influence both awareness and affordability of supplements (12). Saudi studies likewise report variation in supplement use patterns and information sources across different subpopulations and settings (11, 12). Regional disparities may also play a role, as Saudi evidence is largely drawn from city- or region-specific samples alongside national analyses, suggesting heterogeneity by location and population sampled (13, 14). Despite these observations, evidence remains fragmented, and less is known about how demographic, cultural, and digital determinants operate jointly to shape purchasing behavior in Saudi Arabia (11, 13, 15).

Existing research has primarily documented prevalence and product categories in Saudi samples, including community and healthcare-attendant surveys (4, 16, 17) and population-specific assessments of common vitamin/mineral use (18). Across these studies, outcomes are most often reported as prevalence, product type, and selected correlates, whereas purchasing intensity (e.g., purchase frequency or expenditure) and the coexistence of specific trust mechanisms (e.g., online reviews, influencer content, pharmacist counseling) are less frequently examined within a single analytic framework (4, 16, 17). Addressing these gaps is essential for understanding consumer behavior in evolving nutraceutical markets and for guiding strategies in pharmacy practice and regulation.

Accordingly, this study was designed to examine nutraceutical use among adults in Saudi Arabia, with a focus on product hierarchies, purchasing frequency, and the interplay between digital trust sources, professional guidance, and cultural or regulatory signals, while also evaluating the influence of sociodemographic and regional factors.

## 2 Methods

### 2.1 Study design and setting

A cross-sectional, observational online survey was conducted to assess self-reported purchasing and trust mechanisms among members of the general public residing in Saudi Arabia. Data collection ran from May 5, 2025 through mid-August 2025.

### 2.2 Recruitment and dissemination

The survey was administered using Microsoft Forms and disseminated electronically via a shareable link across multiple social media platforms (including both networking and messaging applications). The link was shared as an open invitation; participation was voluntary, and no incentives were offered. To minimize duplicate submissions, Microsoft Forms was configured to restrict responses to one submission per account/device where applicable. In addition, responses were screened during data cleaning for completeness and potential duplication, and incomplete and/or duplicate submissions were removed prior to analysis.

### 2.3 Sample size estimation

The minimum required sample size for this cross-sectional survey was calculated *a priori* using the standard formula for prevalence studies (*n* = *Z*^2^ × *p*[1 − *p*]/*d*^2^), assuming a 95% confidence level (*Z* = 1.96), a conservative expected prevalence of 50%, and a margin of error of 5%. This yielded a minimum required sample of 384 participants. Recruitment was not stopped upon reaching this threshold, and data collection continued throughout the predefined study period to maximize precision and statistical power. The final analytic sample (*n* = 672) exceeded the minimum required sample size.

### 2.4 Eligibility criteria and participant flow

Participants were eligible for inclusion in the analytic sample if they (1) were aged ≥18 years and (2) reported purchasing or consuming any nutraceutical product within the previous 6 months. Eligibility was assessed using the screening item: “In the past six months, have you purchased or consumed any nutraceutical product?”

A total of 1,169 responses were received; 672 met the eligibility criteria and were included in the analysis, while 497 were excluded due to incompleteness/duplication and/or failure to meet eligibility criteria.

### 2.5 Questionnaire development and pilot testing

A self-administered online questionnaire was developed specifically for this study following a comprehensive review of the literature on nutraceutical consumption, trust mechanisms, digital influence, and holistic health practices. The instrument addressed multiple domains, including sociodemographic characteristics, usage patterns, purchasing channels, trust determinants, influencer and social-media impact, holistic health integration, and overall satisfaction. An English version of the final instrument is provided as Supplementary File 1.

Content validity was established through independent review by experts in pharmacy practice and public health, who evaluated the structure, clarity, and relevance of the items. The questionnaire was pilot tested among 20 adult participants to assess comprehension, response consistency, and completion time; minor refinements were made based on feedback. The final version preserved the original screening logic and domain organization and was used for all subsequent data collection and analyses.

### 2.6 Outcome measures and composite scores

Two composite measures were constructed *a priori*. The influencer and social-media impact score was derived by summing two five-point Likert-scale items assessing (1) higher trust when influencer recommendations are evidence-based or reflect long-term personal use, and (2) discovery of nutraceutical products through social-media discussions or testimonials (total range 2–10; higher scores indicate greater influencer and social-media impact). The holistic health integration score was calculated as the sum of two five-point Likert-scale items capturing (1) the integration of nutraceutical use with diet and exercise, and (2) higher trust in products that are halal-certified, clinically tested, or endorsed by Saudi health authorities (range 2–10; higher scores indicate stronger holistic integration). Overall product satisfaction was assessed using a single five-level item.

### 2.7 Data quality

Records failing the screening question were excluded *a priori*. All questionnaire items were mandatory in Microsoft Forms, and only submitted records were retained for analysis. Composite scores were calculated by simple summation of their component items.

### 2.8 Statistical analysis

Descriptive statistics summarized participant characteristics, usage patterns, and item distributions as frequencies and percentages. Bivariable associations between categorical variables were assessed using Pearson’s chi-squared test (or Fisher’s exact test when expected cell counts were small). Purchasing frequency and satisfaction were treated as ordinal outcomes and analyzed using multivariable ordered logistic regression, with results reported as odds ratios (ORs) and 95% confidence intervals (CIs). Composite scores (influencer/social-media impact and holistic health integration; range 2–10) were analyzed using multivariable linear regression and reported as *β* coefficients with 95% CIs. For linear regression models, assumptions were evaluated using residual diagnostics, including assessment of approximate normality and homoscedasticity of residuals. A two-sided *p* value <0.05 was considered statistically significant. Analyses were performed in RStudio 2024.9.1.394 with R 4.4.2.

### 2.9 Ethics

The study received final approval from the Umm Al-Qura University Biomedical Research Ethics Committee (Approval No. HAPO-02-K-012-2025-05-2726; 05 May 2025). The conduct of the research complied with the principles of the Declaration of Helsinki and applicable Saudi national regulations and institutional policies. Participation was voluntary; electronic informed consent was obtained prior to data collection; no direct identifiers were retained; and data were stored on secure, access-restricted servers.

## 3 Results

### 3.1 Sample and characteristics

Of 1,169 responses received, 672 met the inclusion criteria and were included in the analysis, while 497 were excluded due to ineligibility and/or incomplete submissions. The analyzed cohort comprised 431/672 (64.1%) males and 241/672 (35.9%) females. The most frequent age group was 18–29 years: 347/672 (51.6%). Residence was most commonly in the Northern region: 304/672 (45.2%). The highest educational level reported was most often secondary: 347/672 (51.6%). Monthly income most frequently fell in the 10,000 to <20,000 SAR range: 346/672 (51.5%). A very health-conscious dietary self-perception was reported by 253/672 (37.6%). Full distributions are presented in Table 1.

**Table 1:** Demographics and lifestyle characteristics.

Characteristic	Description
Gender	
Male	431 (64.1%)
Female	241 (35.9%)
Age	
18–29	347 (51.6%)
30–39	93 (13.8%)
40–49	96 (14.3%)
50–59	86 (12.8%)
60 or more	50 (7.4%)
Place of residence	
Eastern region	113 (16.8%)
Western region	137 (20.4%)
Northern region	304 (45.2%)
Southern region	61 (9.1%)
Central region	57 (8.5%)
Highest level of education	
Primary	45 (6.7%)
Middle	119 (17.7%)
Secondary	347 (51.6%)
Bachelor	120 (17.9%)
Master	41 (6.1%)
Monthly income (SAR)	
<5,000	104 (15.5%)
5,000 to <10,000	144 (21.4%)
10,000 to <20,000	346 (51.5%)
20,000 or more	78 (11.6%)
Self-perception about dietary habits	
Not at all health-conscious	48 (7.1%)
Not very health-conscious	112 (16.7%)
Neutral	114 (17.0%)
Somewhat health-conscious	145 (21.6%)
Very health-conscious	253 (37.6%)

*n* (%).

### 3.2 Usage patterns and purchasing channels

Product categories and purchasing behaviors were summarized prior to modeling. The most frequently used nutraceutical categories were vitamins and minerals at 554/672 (82.4%), probiotics at 492/672 (73.2%), and herbal or botanical products at 452/672 (67.3%). Purchasing frequency was reported as more than once per month by 252/672 (37.5%), about monthly by 110/672 (16.4%), every 3 months by 123/672 (18.3%), every four to 6 months by 112/672 (16.7%), and rarely by 75/672 (11.2%). Acquisition most commonly occurred through pharmacies or drugstores at 548/672 (81.5%), online retail platforms at 535/672 (79.6%), and brand-specific online stores at 415/672 (61.8%). Detailed distributions are presented in Table 2.

**Table 2:** Usage patterns of nutraceuticals.

Characteristic	Description
Regularly consumed nutraceuticals*	
Vitamins and minerals	554 (82.4%)
Probiotics	492 (73.2%)
Herbal or botanical supplements	452 (67.3%)
Protein powders or functional foods	29 (4.3%)
Omega-3/Essential fatty acids	22 (3.3%)
Frequency of purchasing nutraceuticals	
Rarely	75 (11.2%)
Every 4–6 months	112 (16.7%)
Every 3 months	123 (18.3%)
About once a month	110 (16.4%)
More than once a month	252 (37.5%)
Place of nutraceuticals purchase*	
Pharmacies / Drugstores (e.g., Nahdi/Al-Dawaa)	548 (81.5%)
Online retail platforms (e.g., Amazon.sa/iHerb)	535 (79.6%)
Brand-specific online stores	415 (61.8%)
Health and wellness stores (e.g. GNC)	10 (1.5%)
Supermarkets/Hypermarkets (e.g., Danube/ Carrefour)	13 (1.9%)
Through healthcare practitioners	16 (2.4%)

*n* (%).

*Multiple-response items.

### 3.3 Factors associated with frequent purchasing

Associations between participant characteristics and purchasing frequency were assessed using bivariable analyses and then examined in multivariable models (Table 3). In bivariable comparisons, higher purchasing frequency differed significantly across sex, age group, region of residence, educational level, monthly income, and self-perceived dietary health consciousness (Table 3). In the multivariable ordered logistic regression, higher purchasing frequency remained associated with residence in the Northern versus Eastern region (OR = 1.77, 95% CI 1.11–2.84, *p* = 0.017), monthly income of 10,000 to <20,000 SAR versus <5,000 SAR (OR = 2.07, 95% CI 1.34–3.21, *p* = 0.001), and very health-conscious dietary perception versus the reference category (OR = 12.4, 95% CI 6.31–24.8, *p* < 0.001). In contrast, participants aged 40–49 years (OR = 0.60, 95% CI 0.37–0.96, *p* = 0.034) and 50–59 years (OR = 0.50, 95% CI 0.30–0.81, *p* = 0.005) had lower odds of more frequent purchasing compared with those aged 18–29 years. Adjusted estimates are presented in Table 3.

**Table 3:** Participant characteristics by purchasing frequency and adjusted predictors of higher purchasing frequency.

Characteristic	Frequency of purchasing nutraceuticals						Multivariable analysis*		
	Rarely *N* = 75	Every 4–6 months *N* = 112	Every 3 months *N* = 123	About once a month *N* = 110	More than once a month *N* = 252	*p*-value	OR	95% CI	p-value
Gender						<0.001			
Male	39 (9.0%)	50 (11.6%)	64 (14.8%)	55 (12.8%)	223 (51.7%)		Reference	Reference	
Female	36 (14.9%)	62 (25.7%)	59 (24.5%)	55 (22.8%)	29 (12.0%)		0.79	0.57, 1.10	0.162
Age						<0.001			
18–29	31 (8.9%)	23 (6.6%)	46 (13.3%)	36 (10.4%)	211 (60.8%)		Reference	Reference	
30–39	10 (10.8%)	21 (22.6%)	29 (31.2%)	18 (19.4%)	15 (16.1%)		0.78	0.49, 1.26	0.316
40–49	13 (13.5%)	29 (30.2%)	20 (20.8%)	24 (25.0%)	10 (10.4%)		0.60	0.37, 0.96	**0.034**
50–59	15 (17.4%)	22 (25.6%)	16 (18.6%)	24 (27.9%)	9 (10.5%)		0.50	0.30, 0.81	**0.005**
60 or more	6 (12.0%)	17 (34.0%)	12 (24.0%)	8 (16.0%)	7 (14.0%)		0.63	0.35, 1.16	0.138
Place of residence						<0.001			
Eastern region	21 (18.6%)	25 (22.1%)	26 (23.0%)	24 (21.2%)	17 (15.0%)		Reference	Reference	
Western region	26 (19.0%)	29 (21.2%)	40 (29.2%)	29 (21.2%)	13 (9.5%)		0.66	0.41, 1.06	0.084
Northern region	14 (4.6%)	34 (11.2%)	26 (8.6%)	23 (7.6%)	207 (68.1%)		1.77	1.11, 2.84	**0.017**
Southern region	7 (11.5%)	13 (21.3%)	18 (29.5%)	15 (24.6%)	8 (13.1%)		1.15	0.65, 2.05	0.630
Central region	7 (12.3%)	11 (19.3%)	13 (22.8%)	19 (33.3%)	7 (12.3%)		1.43	0.79, 2.57	0.234
Highest level of education									
Primary	6 (13.3%)	10 (22.2%)	11 (24.4%)	12 (26.7%)	6 (13.3%)		Reference	Reference	
Middle	15 (12.6%)	29 (24.4%)	28 (23.5%)	30 (25.2%)	17 (14.3%)		1.28	0.68, 2.41	0.449
Secondary	27 (7.8%)	35 (10.1%)	37 (10.7%)	35 (10.1%)	213 (61.4%)		1.39	0.75, 2.59	0.290
Bachelor	17 (14.2%)	28 (23.3%)	38 (31.7%)	28 (23.3%)	9 (7.5%)		0.95	0.50, 1.79	0.867
Master	10 (24.4%)	10 (24.4%)	9 (22.0%)	5 (12.2%)	7 (17.1%)		0.65	0.28, 1.47	0.300
Monthly income (SAR)						<0.001			
<5,000	10 (9.6%)	26 (25.0%)	31 (29.8%)	27 (26.0%)	10 (9.6%)		Reference	Reference	
5,000 to <10,000	27 (18.8%)	38 (26.4%)	33 (22.9%)	31 (21.5%)	15 (10.4%)		0.77	0.49, 1.22	0.266
10,000 to <20,000	25 (7.2%)	26 (7.5%)	42 (12.1%)	32 (9.2%)	221 (63.9%)		2.07	1.34, 3.21	**0.001**
20,000 or more	13 (16.7%)	22 (28.2%)	17 (21.8%)	20 (25.6%)	6 (7.7%)		0.66	0.39, 1.12	0.125
Self-perception about dietary habits						<0.001			
Not at all health-conscious	11 (22.9%)	13 (27.1%)	13 (27.1%)	9 (18.8%)	2 (4.2%)		Reference	Reference	
Not very health-conscious	16 (14.3%)	31 (27.7%)	28 (25.0%)	25 (22.3%)	12 (10.7%)		1.40	0.76, 2.62	0.283
Neutral	18 (15.8%)	29 (25.4%)	24 (21.1%)	27 (23.7%)	16 (14.0%)		1.66	0.89, 3.10	0.112
Somewhat health-conscious	24 (16.6%)	30 (20.7%)	46 (31.7%)	29 (20.0%)	16 (11.0%)		1.45	0.80, 2.67	0.224
Very health-conscious	6 (2.4%)	9 (3.6%)	12 (4.7%)	20 (7.9%)	206 (81.4%)		12.4	6.31, 24.8	**<0.001**

*n* (%).

Bivariable comparisons across purchasing-frequency categories were assessed using Pearson’s chi-squared test.

*A multivariable ordered logistic regression model assessing the predictors of high frequency of purchasing nutraceuticals.

CI, Confidence Interval; OR, Odds Ratio. Bold values indicate statistically significant associations (*p* < 0.05).

### 3.4 Online–offline trust, online reviews, and in-store advice

**Figure 1:**
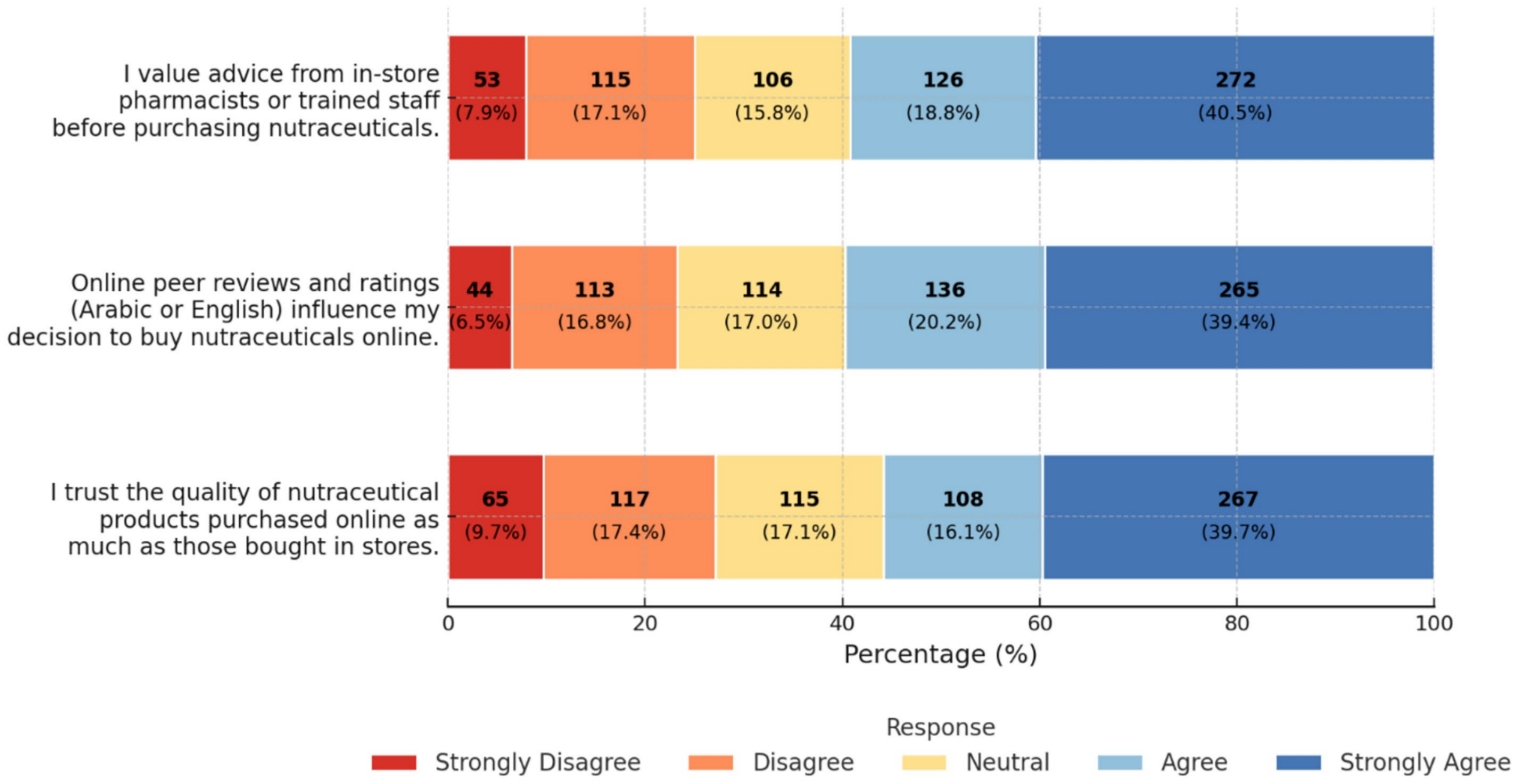
Perceptions and attitudes toward online and offline nutraceutical purchasing and trust. Stacked bar chart of participant responses (%, *n*) across three domains: reliance on in-store advice, influence of online peer reviews, and trust in the quality of online purchases.

Distributions for the three perception items (trust in the quality of online nutraceutical products relative to in-store purchases, influence of online peer reviews on purchasing decisions, and value placed on in-store pharmacist or trained-staff recommendations) are presented in Figure 1. Most participants agreed or strongly agreed that they trust the quality of nutraceutical products purchased online (55.8%) and that online peer reviews influence their purchasing decisions (59.6%). Similarly, 59.3% agreed or strongly agreed that they rely on in-store pharmacist or staff advice before buying nutraceuticals.

In adjusted analyses, purchasing nutraceuticals more than once per month was associated with higher odds of each outcome: trusting the quality of online products compared with in-store purchases (OR 5.18; 95% CI 2.80–9.65; *p* < 0.001), being influenced by online peer reviews (OR 4.34; 95% CI 2.31–8.22; *p* < 0.001), and valuing in-store pharmacist or staff advice (OR 3.02; 95% CI 1.65–5.56; *p* < 0.001). Very health-conscious participants were also more likely to value in-store advice (OR 3.67; 95% CI 1.79–7.60; *p* < 0.001). Participants from the Northern region were more likely than those from the Eastern region to trust online products (OR 1.98; 95% CI 1.21–3.23; *p* = 0.006) and to value pharmacist recommendations (OR 1.77; 95% CI 1.11–2.84; *p* = 0.017). A similar pattern was observed in the Western region for valuing in-store advice (OR 1.65; 95% CI 1.03–2.65; *p* = 0.039). Female sex remained associated with lower odds across online-trust outcomes, and older age groups showed reduced likelihood of reporting online trust, influence of reviews, and reliance on pharmacist advice. Full adjusted estimates are presented in Table 4.

**Table 4:** Predictors of trust in online nutraceutical products, influence on the decision to buy nutraceuticals online and recommendations of in-store pharmacists or trained staff.

Characteristic	Trust in online nutraceutical products			Influence on the decision to buy nutraceuticals online			Recommendations of in-store pharmacists or trained staff		
	OR	95% CI	*p*-value	OR	95% CI	*p*-value	OR	95% CI	*p*-value
Gender									
Male	Reference	Reference		Reference	Reference		Reference	Reference	
Female	0.56	0.40, 0.79	**<0.001**	0.44	0.31, 0.62	**<0.001**	0.65	0.46, 0.90	**0.010**
Age									
18–29	Reference	Reference		Reference	Reference		Reference	Reference	
30–39	0.59	0.36, 0.96	**0.034**	0.40	0.24, 0.65	**<0.001**	0.59	0.36, 0.96	**0.032**
40–49	0.43	0.27, 0.70	**<0.001**	0.36	0.22, 0.59	**<0.001**	0.46	0.28, 0.76	**0.003**
50–59	0.47	0.29, 0.78	**0.004**	0.49	0.30, 0.80	**0.004**	0.57	0.35, 0.93	**0.024**
60 or more	0.88	0.48, 1.61	0.678	0.66	0.36, 1.21	0.176	0.33	0.18, 0.58	**<0.001**
Place of residence									
Eastern region	Reference	Reference		Reference	Reference		Reference	Reference	
Western region	0.85	0.53, 1.37	0.508	1.00	0.62, 1.61	0.995	1.65	1.03, 2.65	0.039
Northern region	1.98	1.21, 3.23	**0.006**	1.22	0.75, 1.98	0.415	1.77	1.11, 2.84	**0.017**
Southern region	0.60	0.34, 1.08	**0.088**	1.69	0.95, 3.01	0.073	0.83	0.46, 1.48	0.530
Central region	0.76	0.43, 1.34	0.346	0.89	0.48, 1.63	0.697	0.89	0.50, 1.61	0.705
Highest level of education									
Primary	Reference	Reference		Reference	Reference		Reference	Reference	
Middle	1.31	0.70, 2.46	0.391	1.45	0.79, 2.70	0.234	1.35	0.73, 2.50	0.337
Secondary	1.48	0.81, 2.70	0.205	3.15	1.72, 5.78	**<0.001**	2.63	1.45, 4.78	**0.001**
Bachelor	1.23	0.66, 2.32	0.515	1.84	1.00, 3.42	0.052	2.39	1.29, 4.46	**0.006**
Master	0.74	0.34, 1.64	0.464	0.94	0.43, 2.07	0.879	1.18	0.56, 2.49	0.660
Monthly income (SAR)									
<5,000	Reference	Reference		Reference	Reference		Reference	Reference	
5,000 to <10,000	1.15	0.73, 1.83	0.548	1.34	0.84, 2.14	0.217	0.93	0.58, 1.49	0.774
10,000 to <20,000	1.53	0.98, 2.41	0.064	2.23	1.41, 3.52	**<0.001**	1.54	0.97, 2.42	0.064
20,000 or more	0.93	0.54, 1.61	0.805	1.62	0.94, 2.79	0.082	0.89	0.52, 1.53	0.683
Self-perception about dietary habits									
Not at all health-conscious	Reference	Reference		Reference	Reference		Reference	Reference	
Not very health-conscious	0.80	0.42, 1.53	0.507	0.75	0.40, 1.42	0.381	1.38	0.73, 2.59	0.320
Neutral	0.81	0.42, 1.54	0.521	0.65	0.34, 1.23	0.182	1.24	0.67, 2.32	0.496
Somewhat health-conscious	0.59	0.31, 1.11	0.101	0.76	0.41, 1.40	0.378	1.43	0.77, 2.65	0.259
Very health-conscious	1.85	0.90, 3.81	0.092	1.83	0.91, 3.69	0.088	3.67	1.79, 7.60	**<0.001**
Frequency of purchasing nutraceuticals									
Rarely	Reference	Reference		Reference	Reference		Reference	Reference	
Every 4–6 months	1.02	0.58, 1.77	0.950	1.55	0.91, 2.66	0.110	1.27	0.74, 2.16	0.384
Every 3 months	0.82	0.48, 1.40	0.472	0.76	0.44, 1.30	0.314	0.86	0.51, 1.46	0.583
About once a month	0.78	0.45, 1.36	0.381	1.10	0.64, 1.91	0.726	0.94	0.55, 1.60	0.825
More than once a month	5.18	2.80, 9.65	**<0.001**	4.34	2.31, 8.22	**<0.001**	3.02	1.65, 5.56	**<0.001**

OR, Odds Ratio; CI, Confidence Interval.

Bold values indicate statistically significant associations (*p* < 0.05).

### 3.5 Influencer or social-media impact, holistic integration, and satisfaction

**Figure 2:**
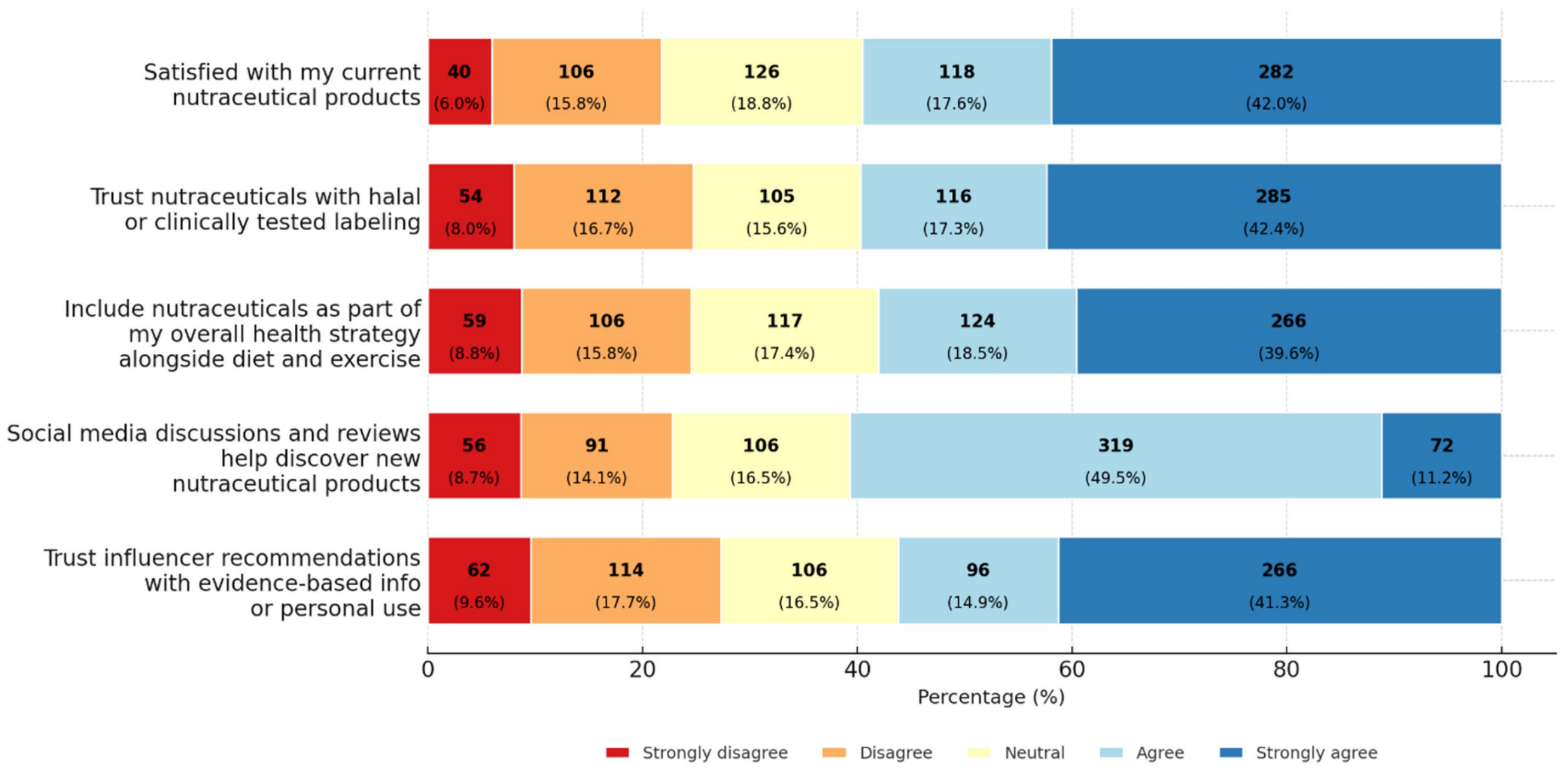
Perceptions and attitudes toward nutraceuticals and social media influence. Stacked bar chart of participant responses (*n*, %) across five domains: influencer trust, social media product discovery, health strategy integration, trust in certified products, and satisfaction.

Item-level distributions for influencer and social media impact, holistic health integration, and satisfaction are summarized in Figure 2. Following Saudi or regional health influencers or online communities was reported by 439 of 672 participants (65.3%). Agreement that evidence-based or long-term-use influencer content increases trust was 362 of 672 (56.2%), and discovering new products through social media discussions or testimonials was 408 of 672 (60.7%). Holistic integration of nutraceuticals with diet and exercise was reported by 391 of 672 (58.1%), and trust based on halal certification, clinical testing, or official endorsement was 401 of 672 (59.7%). Overall satisfaction with currently used products was 401 of 672 (59.6%).

In multivariable models (Table 5), higher influencer-impact scores were associated with very health-conscious status (*β* 1.39; 95% CI 0.63–2.14; *p* < 0.001), monthly income of 10,000 to <20,000 SAR (*β* 0.88; 95% CI 0.38–1.38; *p* < 0.001), and purchasing nutraceuticals more than once per month (*β* 1.57; 95% CI 0.92–2.23; *p* < 0.001) or every 3 months (*β* 0.71; 95% CI 0.12–1.30; *p* = 0.018). Higher holistic-integration scores were observed among very health-conscious participants (*β* 1.10; 95% CI 0.50–1.70; *p* < 0.001), those residing in the Northern region (*β* 0.51; 95% CI 0.09–0.92; *p* = 0.017), and those purchasing more than once per month (*β* 0.74; 95% CI 0.22–1.26; *p* = 0.005). Lower holistic-integration scores were found among females (*β* −0.42; 95% CI −0.71 to −0.13; *p* = 0.004) and among participants aged 30–59 years compared with those aged 18–29 years.

**Table 5:** Results of the predictors of high score of impact of influencers and social media and holistic health integration as well as the predictors of satisfaction regarding the currently used nutraceutical products.

Characteristic	High score of impact of influencers and social media^#^			High score of holistic health integration^#^			Satisfaction level regarding the currently used nutraceutical products*		
	Beta	95% CI	*p*-value	Beta	95% CI	*p*-value	OR	95% CI	*p*-value
Gender									
Male	Reference	Reference		Reference	Reference		Reference	Reference	
Female	−0.30	−0.67, 0.06	0.101	−0.42	−0.71, −0.13	0.004	0.71	0.51, 0.99	**0.043**
Age									
18–29	Reference	Reference		Reference	Reference		Reference	Reference	
30–39	0.26	−0.28, 0.79	0.343	−1.21	−1.63, −0.79	<0.001	0.61	0.38, 1.00	0.051
40–49	−0.10	−0.63, 0.43	0.719	−0.90	−1.32, −0.48	<0.001	0.40	0.24, 0.66	**<0.001**
50–59	0.49	−0.05, 1.03	0.077	−1.12	−1.55, −0.69	<0.001	0.36	0.22, 0.58	**<0.001**
60 or more	0.63	−0.03, 1.29	0.063	−0.72	−1.24, −0.20	0.007	1.19	0.64, 2.22	0.575
Place of residence									
Eastern region	Reference	Reference		Reference	Reference		Reference	Reference	
Western region	−0.34	−0.86, 0.17	0.193	0.09	−0.32, 0.50	0.672	1.42	0.89, 2.27	0.137
Northern region	−0.02	−0.54, 0.51	0.953	0.51	0.09, 0.92	0.017	1.83	1.13, 2.97	**0.014**
Southern region	−0.63	−1.27, 0.00	0.050	−0.17	−0.67, 0.34	0.513	1.36	0.75, 2.46	0.305
Central region	−0.52	−1.17, 0.13	0.116	0.12	−0.39, 0.64	0.637	1.80	1.00, 3.26	0.050
Highest level of education									
Primary	Reference	Reference		Reference	Reference		Reference	Reference	
Middle	0.06	−0.63, 0.75	0.868	0.26	−0.29, 0.81	0.347	0.71	0.37, 1.36	0.304
Secondary	0.04	−0.63, 0.71	0.911	0.68	0.15, 1.21	0.012	1.35	0.73, 2.52	0.339
Bachelor	−0.14	−0.84, 0.55	0.685	0.41	−0.15, 0.96	0.149	0.94	0.49, 1.79	0.842
Master	0.21	−0.65, 1.07	0.637	−0.03	−0.71, 0.66	0.941	0.51	0.23, 1.12	0.094
Monthly income (SAR)									
<5,000	Reference	Reference		Reference	Reference		Reference	Reference	
5,000 to <10,000	0.27	−0.24, 0.78	0.303	−0.38	−0.79, 0.03	0.066	0.97	0.61, 1.54	0.887
10,000 to <20,000	0.88	0.38, 1.38	**<0.001**	0.33	−0.07, 0.73	0.102	1.79	1.13, 2.82	**0.013**
20,000 or more	0.28	−0.32, 0.88	0.363	−0.02	−0.49, 0.46	0.936	0.56	0.32, 0.97	**0.039**
Self-perception about dietary habits									
Not at all health-conscious	Reference	Reference		Reference	Reference		Reference	Reference	
Not very health-conscious	0.31	−0.38, 1.00	0.383	0.38	−0.17, 0.93	0.176	0.66	0.34, 1.25	0.197
Neutral	−0.05	−0.75, 0.64	0.878	0.25	−0.30, 0.80	0.380	0.65	0.34, 1.23	0.181
Somewhat health-conscious	−0.08	−0.76, 0.59	0.810	0.25	−0.29, 0.78	0.363	1.19	0.64, 2.23	0.589
Very health-conscious	1.39	0.63, 2.14	**<0.001**	1.10	0.50, 1.70	<0.001	3.93	1.92, 8.08	**<0.001**
Frequency of purchasing nutraceuticals									
Rarely	Reference	Reference		Reference	Reference		Reference	Reference	
Every 4–6 months	0.60	0.00, 1.20	0.050	−0.49	−0.96, −0.01	0.045	1.01	0.58, 1.77	0.968
Every 3 months	0.71	0.12, 1.30	**0.018**	0.03	−0.43, 0.50	0.896	0.70	0.41, 1.20	0.193
About once a month	0.23	−0.37, 0.84	0.445	−0.22	−0.70, 0.26	0.364	0.77	0.45, 1.34	0.360
More than once a month	1.57	0.92, 2.23	**<0.001**	0.74	0.22, 1.26	0.005	2.43	1.30, 4.54	**0.005**

^#^Results are based on multivariable linear regression models.

*A multivariable ordered logistic regression model assessing the satisfaction level regarding the currently used nutraceutical products.

CI, Confidence Interval; OR, Odds Ratio.

Bold values indicate statistically significant associations (*p* < 0.05).

Greater satisfaction with currently used nutraceuticals was associated with being very health-conscious (OR 3.93; 95% CI 1.92–8.08; *p* < 0.001), residence in the Northern (OR 1.83; 95% CI 1.13–2.97; *p* = 0.014) or Central (OR 1.80; 95% CI 1.00–3.26; *p* = 0.050) regions, income of 10,000 to <20,000 SAR (OR 1.79; 95% CI 1.13–2.82; *p* = 0.013), and purchasing more than once per month (OR 2.43; 95% CI 1.30–4.54; *p* = 0.005). Female participants and those aged 40 to 59 years reported lower satisfaction compared with the 18–29 age group.

## 4 Discussion

This study examined nutraceutical use, purchasing channels, and trust mechanisms among adults in Saudi Arabia. Among 672 recent users, vitamins and minerals were the most common products, followed by probiotics and herbal or botanical supplements. Purchasing was often more than once per month, and higher frequency was associated with very high health consciousness, mid-tier income, and residence in the Northern region. These purchasing behaviors should be viewed within the context of a rapidly expanding nutraceutical market in the Gulf region, which is projected to reach USD 22 billion by 2025 (5), and in light of changes following the COVID-19 pandemic that strengthened preventive health practices and increased supplement use (3). Digital trust mechanisms, including online peer reviews and influencer content, coexisted with traditional validation through pharmacist or staff advice, while halal certification, clinical testing, and official endorsement remained important quality cues. Together, these findings highlight a hybrid consumer environment in which digital influence, cultural norms, and professional guidance interact, and these elements were associated with participants’ self-reported behaviors and perceptions. This pattern mirrors global trends showing that health maintenance and disease prevention continue to drive supplement adoption across diverse populations (1, 2).

The leading role of vitamins and minerals, reported by 554 of 672 participants (82.4%), aligns with prior Saudi and GCC surveys where vitamin-based products consistently dominate (4, 16–18). Probiotics, reported by 492 of 672 (73.2%), and herbal or botanical products, by 452 of 672 (67.3%), were also common, reflecting the digitally mediated spread of information and the culturally embedded preference for natural remedies (1, 2). This distribution may relate to differential familiarity with product categories and variation in salient trust cues across purchasing channels, including pharmacist counseling and visible quality markers (10). Another contributing factor may be increased attention to digestive health, which has elevated probiotic use (3), alongside enduring herbal traditions that sustain botanical use, with online promotion potentially increasing the salience of both product types (7, 19). The prominence of probiotics and botanicals is also consistent with heightened interest in immune support and natural health approaches during and after the COVID-19 period (3, 8). This product hierarchy provides a foundation for understanding purchasing intensity and its correlates.

Purchasing cadence concentrated in the upper ranges: more than once per month in 252 of 672 (37.5%), monthly in 110 of 672 (16.4%), every 3 months in 123 of 672 (18.3%), every 4–6 months in 112 of 672 (16.7%), and rarely in 75 of 672 (11.2%). Very health-conscious respondents had markedly higher odds of frequent purchasing (OR 12.4; 95% CI 6.31–24.8; *p* < 0.001), with a larger magnitude than regional associations reported in prior work for any use (OR 3.5, 2.1–5.8; OR 2.1, 1.4–3.0; *β* 0.5, 0.2–0.8) (15, 20). This contrast may reflect differences in construct definition and outcome specification, as our top-coded “very” category, an outcome of purchasing frequency rather than prevalence, and a user-only denominator are likely to accentuate effect sizes. Residual confounding may also contribute, including socioeconomic correlates such as income (21). Accordingly, purchasing intensity is best interpreted here as a behavioral marker of engagement rather than an indicator of clinical benefit, as effectiveness, safety outcomes, and health endpoints were not assessed in this survey. These findings also need to be considered in the context of rapid market expansion, with the GCC nutraceutical sector projected to reach USD 22 billion by 2025 (5). In such an environment, purchasing frequency may reflect not only individual orientations but also structural drivers of availability, marketing intensity, and consumer spending capacity. Purchasing intensity therefore sheds light on how trust is constructed within this subgroup.

Frequent purchasers more often endorsed online products as equivalent to in-store options, reported influence from peer reviews, and simultaneously valued pharmacist or trained-staff advice. These associations, which remained significant across all outcomes, suggest complementarity rather than substitution. Digital channels expand exposure and provide social validation (22), whereas pharmacists may strengthen confidence by confirming product quality, clarifying interactions, and contextualizing use (23, 24). This pattern is consistent with high social media engagement and the enduring professional authority of pharmacists in Saudi Arabia. The coexistence of these mechanisms suggests a layered trust structure, whereby digital reviews and influencer content may facilitate product discovery and relatability, while pharmacists may provide evidence-informed contextualization and risk communication. Prior work has underscored pharmacists’ capacity to correct misinformation and moderate commercial narratives, indicating that models incorporating pharmacist input into digital health communication warrant evaluation for their potential to support informed nutraceutical decision-making (7, 10).

Social media was also a common discovery route, reported by 408 of 672 participants (60.7%), and 362 of 672 (56.2%) agreed that evidence-based or long-term-use claims increase trust. External studies confirm stronger effects when influencer content references evidence (*β* ≈ 0.34–0.45) than when it is purely anecdotal (*β* ≈ 0.30 or non-significant) (7, 25, 26). This credibility gradient is consistent with the notion that higher-cost signals, such as explicit evidence or sustained personal use, may be perceived as more persuasive than low-cost anecdotal cues (27). The distinction may contribute to weaker or inconsistent effects when influencer narratives lack verifiable anchors, particularly in health contexts where risk perception is salient. Conversely, when influencers integrate credible information or long-term adherence into their messaging, trust may be more strongly aligned with cues associated with professional or scientific endorsement. Accordingly, approaches that incorporate evidence-based input into digital health communication, including through collaboration with pharmacists or qualified experts, warrant evaluation for feasibility, accuracy, and impact.

Codified quality marks also appeared to reinforce trust. Halal certification, clinical testing, or authority endorsement increased confidence for 401 of 672 participants (59.7%). In other Muslim-majority settings, agreement with halal labeling alone ranges from 68 to 83% (8, 28–30). The slightly lower proportion observed here likely reflects the combination of three quality signals into a single item and the focus on recent users. Even so, verifiable marks may serve as salient anchors of trust, and their clear presentation in both online and physical retail contexts may support consumer confidence (31). Future studies could disentangle the individual effects of halal, clinical, and regulatory signals to better quantify their relative influence on consumer decision-making. The emphasis on these quality marks also aligns with the broader regulatory environment. Relative to pharmaceuticals, nutraceutical oversight is often perceived as less transparent, which may be associated with greater reliance on visible certifications and professional guidance as practical cues of quality. Accordingly, initiatives that improve labeling clarity, product verification, and substantiation of claims warrant evaluation for their potential to support informed choice and confidence across consumer groups (9, 10).

At the same time, these patterns intersect with gender and behavioral differences. Although women are generally more likely to engage with complementary and alternative medicine, including nutraceuticals (32–34), the present findings indicate lower holistic integration among female participants. This contrast suggests that while awareness and interest may be high, the ways in which nutraceutical use is integrated with broader health practices may differ across groups. Such divergence may relate to differences in perceived risk, information sources, or practical constraints, including competing responsibilities, though these pathways were not directly assessed in the present study (25, 35, 36).

Mid-income respondents demonstrated higher purchasing frequency and greater responsiveness to influencer cues. Behavioral evidence suggests that this group is particularly elastic to explicit value and quality signals, with attenuation observed in habitual categories or with familiar brands (21, 37–40). This pattern is consistent with economic perspectives in which mid-income groups often exhibit greater sensitivity to transparent quality or price indicators than higher-income consumers, who may rely more on brand familiarity, or lower-income consumers, who may face affordability constraints. Regionally, Northern residence was associated with more frequent purchasing (OR 1.77; 95% CI 1.11–2.84; *p* = 0.017). This association may reflect contextual differences such as retail access, cultural preferences, or patterns of digital engagement, and it warrants further investigation to clarify the underlying drivers of these regional variations.

## 5 Limitations

The interpretation of these findings may be influenced by several considerations. The cross-sectional design limits the ability to infer causality or establish temporal relationships between determinants and outcomes. Recruitment through an online convenience approach disseminated via social media means the sampling frame and response rate are not precisely defined; selection and self-selection bias may therefore be present, and generalizability to the wider adult population in Saudi Arabia may be constrained. In addition, the measures relied on self-report and may be affected by recall or social desirability bias. The self-administered format also did not allow interviewer-led clarification of survey items, which could have influenced how some questions were understood. Although analyses adjusted for key sociodemographic variables, residual confounding from unmeasured factors may remain. Adverse events and safety outcomes were not assessed in this survey, and the study did not evaluate the clinical effectiveness or health benefits of nutraceutical use.

## 6 Conclusion

In this nationwide cross-sectional online survey of adults in Saudi Arabia reporting nutraceutical use within the previous 6 months, purchasing patterns and reported trust mechanisms were associated with digital platforms, cultural practices, and sociodemographic factors. Vitamins and minerals were commonly reported, while probiotics and botanical products may reflect both digitally mediated diffusion and established health-related beliefs. Trust appeared layered, with online reviews and influencer-related content supporting discovery and social validation, pharmacist or trained-staff counseling providing professional reassurance, and perceived quality cues such as halal certification or regulatory endorsement contributing to confidence. Women reported lower holistic integration and satisfaction, which may indicate gender-related differences in engagement and perceived value. Overall, these findings suggest that strengthening the clarity and verifiability of product information, encouraging responsible digital marketing practices, and supporting access to pharmacist counseling may contribute to more informed nutraceutical decision-making within an increasingly hybrid marketplace.

## Data Availability

The raw data supporting the conclusions of this article will be made available by the authors without undue reservation.
