# Supplementary material for: Patterns and determinants of nutraceutical use and trust mechanisms among adults in Saudi Arabia: a cross-sectional study
Source: Front Med (Lausanne). 2026 Apr 1;13:1779602. doi: 10.3389/fmed.2026.1779602 (PMC13078981; doi:10.3389/fmed.2026.1779602)
Supplement: Supplementary file 1 [file Table_1.DOCX]

**Supplementary File 1. English Translation of the Final Study Questionnaire**

**Title:**
*Patterns and Determinants of Nutraceutical Use and Trust Mechanisms Among Adults in Saudi Arabia: A Cross-Sectional Study*

**Section 1. Screening**

1. In the past six months, have you purchased or consumed any nutraceutical or health supplement product (e.g., vitamins, probiotics, herbal extracts, dietary supplements)?
    ☐ Yes → Continue
    ☐ No → End of survey

**Section 2. General Information**

1. Gender
    ☐ Male ☐ Female
2. Age group
    ☐ 18–24 ☐ 25–34 ☐ 35–44 ☐ 45–54 ☐ 55–64 ☐ 65 or above
3. Region of residence (Saudi Arabia)
    ☐ Central ☐ Western ☐ Eastern ☐ Northern ☐ Southern
4. Highest educational level completed
    ☐ High school or below ☐ Diploma / Some college ☐ Bachelor’s degree ☐ Master’s degree or higher
5. Monthly household income (SAR)
    ☐ <5,000 ☐ 5,000–9,999 ☐ 10,000–19,999 ☐ ≥20,000 ☐ Prefer not to say
6. How would you describe your dietary habits?
    ☐ Very health-conscious ☐ Somewhat health-conscious ☐ Neutral ☐ Not very health-conscious ☐ Not at all health-conscious

**Section 3. Patterns of Nutraceutical Use**

1. What types of nutraceuticals do you regularly consume? (Select all that apply)
    ☐ Vitamins and minerals ☐ Probiotics ☐ Herbal/botanical products ☐ Omega-3 or essential fatty acids ☐ Protein powders or functional foods ☐ Other: ________
2. How often do you purchase nutraceuticals?
    ☐ More than once a month ☐ About once a month ☐ Every 2–3 months ☐ A few times a year ☐ Rarely
3. Where do you primarily purchase these products? (Select all that apply)
    ☐ Online retail platforms (e.g., Amazon.sa, iHerb) ☐ Brand-specific online stores ☐ Pharmacies or drugstores ☐ Health and wellness stores ☐ Supermarkets / Hypermarkets ☐ Other: ________

**Section 4. Online vs. In-Store Trust**

*(1 = Strongly disagree; 5 = Strongly agree)*

1. I trust the quality of nutraceutical products purchased online as much as those bought in physical stores.
2. Reading online peer reviews influences my decision to buy nutraceuticals.
3. I value the advice of in-store pharmacists or trained staff before buying nutraceuticals.

**Section 5. Influencer and Social Media Impact (Screening)**

1. Do you follow any social-media influencers, bloggers, or online communities focused on health or nutrition?
    ☐ Yes ☐ No

(If “No,” skip to Section 7)

**Section 6. Influencer and Social Media Impact (Attitudes)**

*(1 = Strongly disagree; 5 = Strongly agree)*

1. I trust information and recommendations provided by influencers about health and wellness, especially when based on evidence or genuine long-term use.
2. Influencer recommendations and social-media discussions influence my decisions to discover or purchase nutraceutical products.

**Section 7. Holistic Health Integration and Satisfaction**

*(1 = Strongly disagree; 5 = Strongly agree, except where noted)*

1. I use nutraceutical products as part of my personal approach to maintaining or improving my health, along with diet, exercise, and other wellness practices.
2. I have greater trust in nutraceuticals that are certified, recommended by health authorities, or advised by a trusted health professional.
3. Overall, how satisfied are you with the nutraceutical products you currently use?
    ☐ Very satisfied ☐ Somewhat satisfied ☐ Neutral ☐ Somewhat dissatisfied ☐ Very dissatisfied
